# Modification and Potential Application of Short-Chain-Length Polyhydroxyalkanoate (SCL-PHA)

**DOI:** 10.3390/polym8080273

**Published:** 2016-07-28

**Authors:** Shichao Wang, Wei Chen, Hengxue Xiang, Junjie Yang, Zhe Zhou, Meifang Zhu

**Affiliations:** State Key Laboratory for Modification of Chemical Fibers and Polymer Materials, College of Materials Science and Engineering, Donghua University, Shanghai 201620, China; wangscdhu@163.com (S.W.); Ava_1227xxi@163.com (W.C.); yangjunjie@mail.dhu.edu.cn (J.Y.); zzhe@dhu.edu.cn (Z.Z.)

**Keywords:** polyhydroxyalkanoate (PHA), modification, crystallization behavior, thermal stability, mechanical property

## Abstract

As the only kind of naturally-occurring biopolyester synthesized by various microorganisms, polyhydroxyalkanoate (PHA) shows a great market potential in packaging, fiber, biomedical, and other fields due to its biodegradablity, biocompatibility, and renewability. However, the inherent defects of scl-PHA with low 3HV or 4HB content, such as high stereoregularity, slow crystallization rate, and particularly the phenomena of formation of large-size spherulites and secondary crystallization, restrict the processing and stability of scl-PHA, as well as the application of its products. Many efforts have focused on the modification of scl-PHA to improve the mechanical properties and the applicability of obtained scl-PHA products. The modification of structure and property together with the potential applications of scl-PHA are covered in this review to give a comprehensive knowledge on the modification and processing of scl-PHA, including the effects of physical blending, chemical structure design, and processing conditions on the crystallization behaviors, thermal stability, and mechanical properties of scl-PHA.

## 1. Introduction

**Figure 1:**
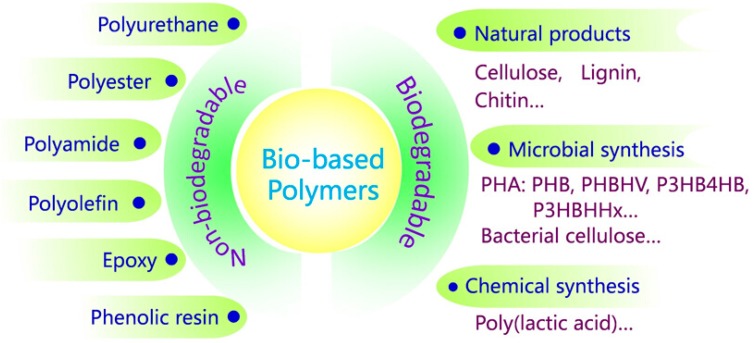
Classification of bio-based polymers.

Bio-based polymers are produced from biomass resources using biological, physical, or chemical methods. According to their biodegradability, bio-based polymers can be divided into two broad categories, namely biodegradable and non-biodegradable polymers [1]. As shown in Figure 1, biodegradable polymers, including cellulose, lignin, and chitin, can be produced directly from plants and animals using physical or chemical methods. Alternatively, biodegradable polymers can be obtained by biological fermentation [2] (such as PHA, bacterial cellulose) or chemical synthesis (such as poly(lactic acid)). Non-biodegradable polymers synthesized partially from biomass include polyurethane, polyester, polyamide 56, polyolefin, epoxy, and phenolic resin. Renewability and biocompatibility make bio-based polymers attractive as green materials and these polymers also have potential applications in the biomedical field [3,4,5]. 

**Figure 2:**
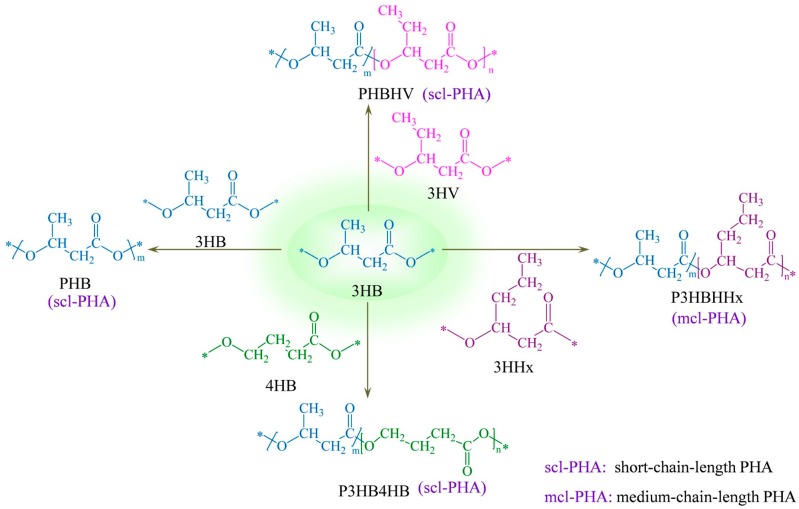
General structure of various copolymers of PHA.

Bio-based polymers have attracted increasing attention over the last two decades, predominantly due to environmental concerns and the realization of limited oil reserves [6]. Among all the bio-based polymers, PHA has received even more interest due to its biodegradability, biocompatibility, chemical diversity, and manufacture from renewable carbon resources by biological fermentation [7,8,9]. In 1926, Lemoigne first discovered and extracted polyhydroxybutyrate (P3HB) homo-polymer from microorganisms [10]. However, many disadvantages, including the poor thermal stability, high brittleness, and narrow processing temperature window, restricted the development of P3HB until the 1970s, when the petroleum crisis and environmental pollution concerns arose [11]. Since the physical and chemical properties of PHA are similar to those of common petroleum-based plastics such as polypropylene, PHA is expected to be able to partially replace petroleum-based polymers, and to reduce environment pollution at the same time [9]. With the extensive study of PHA, more than 150 kinds of building blocks of PHA with different structures and special performances have been reported [12]. All of the developed PHAs are linear polyesters and their basic chemical structure can be roughly expressed as 
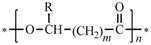
 (*m* = 1–3), where *n* is the degree of polymerization, and *R* is the alkyl group with different chain lengths and structures [13,14]. After poly(3-hydroxybutyrate) (P3HB), poly(3-hydroxybutyrate-*co*-3-hydroxyvalerate) (PHBHV) and poly(3-hydroxybutyrate-*co*-4-hydroxybutyrate) (P3HB4HB) were produced by Imperial Chemical Industries (ICI) and Metabolix Inc. in 1981 and 1992, respectively [15]. Poly(3-hydroxybutyrate-*co*-3-hydroxyhexanoate) (PHBHHx) was successfully synthesized in China seven years later. Figure 2 shows the general structural formula of various copolymers of PHA. According to the number of carbon atoms in the monomer, PHA can be divided into short-chain-length PHA (scl-PHA), medium-chain-length PHA (mcl-PHA), and long-chain-length PHA (lcl-PHA), corresponding to 3–5, 6–14, and 15 or more carbon atoms, respectively [16]. 

Compared with mcl-PHA, scl-PHA has been extensively investigated, partially due to its high production. The largest producer of scl-PHA is Tianan Biologic Materials Co., Ltd. (Ningbo, China), with an annual PHBHV production capacity of 2000t. However, the inherent structure defects of scl-PHA with low 3HV or 4HB content, including the high stereoregularity, slow crystallization rate, and the phenomena of formation of large-size spherulites and secondary crystallization, limit its further application in the packaging, textile, and biomaterial fields. To solve the problems mentioned above, methods such as physical blending and chemical structure design combined with processing conditions have been applied to improve the mechanical properties and applicability of scl-PHA [17].

## 2. Modification of scl-PHA

### 2.1. Basic Properties of scl-PHA

#### 2.1.1. Crystal Structure and Crystallization Behavior of scl-PHA

The crystal structures of P3HB and P3HV belong to the orthogonal crystal system [18] with cell parameters of (*a* = 0.576 nm, *b* = 1.320 nm, *c* = 0.596 nm) and (*a* = 0.932 nm, *c* = 0.556 nm, *b* = 1.002 nm) [19], respectively. As a copolyester of P3HB and P3HV, PHBHV is a kind of chiral polymer with a high degree of optical rotation. The crystal structure and *d* spacing of PHBHV with 0–95 mol % HV content have been studied with the help of X-ray diffraction by Doi et al. [20]. As shown in Table 1, PHBHV presented a P3HB crystal structure when HV content was below 37 mol %. With the increasing content of HV, the *d* spacing of (110) plane increased significantly, while those of the (020) and (002) planes remained unchanged, indicating that the side chains of HV units expanded the (110) plane of the P3HB lattice due to the steric effect. When the content of HV was between 53 and 95 mol %, the crystal structure of PHBHV was similar to that of P3HV, and no significant change was found in all the *d* spacings of the P3HV lattice [20]. Surprisingly, the coexistence of both crystal phases and pseudoeutectic behavior of P3HB and P3HV occurred at a narrow composition range and an isodimorphism phenomenon was formed in PHBHV with 40 mol % HV content [21,22,23,24]. Similar to PHBHV, the crystallization mechanism and crystal structure of P3HB4HB were similar to those of P3HB. The crystallization rate and the *d* spacing were decreased while the radius of spherulite increased with the increasing content of 4HB [25].

**Figure 3:**
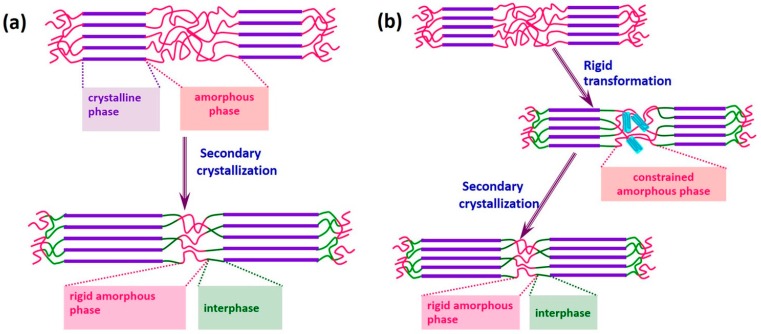
Brittle evolution mechanism for PHBHV: (**a**) secondary crystallization; (**b**) rigid transformation for amorphous phase.

With the increase of storage time, scl-PHA usually experiences a transition process from viscoelasticity to brittleness. Normally, the secondary crystallization is considered to be the main reason for the transition of scl-PHA. Figure 3 describes the brittle evolution mechanism for PHBHV. As shown in Figure 3a, the amorphous region is firstly bounded by the crystalline region after the formation of PHBHV products. Then, the interphase and rigid amorphous phase are formed in the amorphous region by the effect of secondary crystallization. As a result, the scl-PHA products become relatively brittle. In our previous work, we found that before the secondary crystallization developed to a large degree, PHBHV still had a large elongation after heat drawing treatment. Therefore, we propose that the brittleness of PHBHV obeys the rigid transformation of amorphous phase, as shown in Figure 3b. The amorphous region can be frozen by rigid transformation due to the intermolecular interaction or a change in environmental conditions, leading to a constrained amorphous phase. Unlike the abovementioned rigid amorphous phase, this constrained structure can be rearranged under a certain temperature. With the further development of secondary crystallization, the amorphous region will be entirely bounded by the crystalline region and will not be able to undertake a tensile deformation even at a high temperature.

#### 2.1.2. Thermal Properties of scl-PHA

##### The Multiple Melting Behavior of scl-PHA

The state of amorphous, semi-crystalline, or crystalline can be found in semi-crystallined scl-PHA according to the differences in chemical structure, molecular weight, and processing conditions. The double or multiple melting behavior of scl-PHA has been observed with the help of DSC [26]. Normally, the multiple melting behavior of scl-PHA is related to the annealing condition, the heating rate, and the structure and morphology of crystals. The formation mechanism of the multiple melting behavior of scl-PHA has been intensively studied, and the identified mechanisms are listed as follows: (1) the melt–recrystallization–remelt process; (2) the melting process of different crystals; (3) the melting process of lamellas with variety of morphologies; (4) the melting process of different molecular weights; and (5) the physical aging and/or relaxation of the rigid amorphous fraction [21]. In order to describe the melting behavior of P3HB in a more accurate way, Wellen et al. systematically investigated the first melt behavior and its repeatability of P3HB using DSC. The analysis of DSC energy flow scans showed a complex melting peak that can be resolved into three elementary peaks with different intensities at different melting temperatures. Results suggested that the peak temperature of P3HB depended on the heating rate, while the total crystallinity detected was independent of the heating rate [27]. 

##### Thermal Stability of scl-PHA

**Figure 4:**
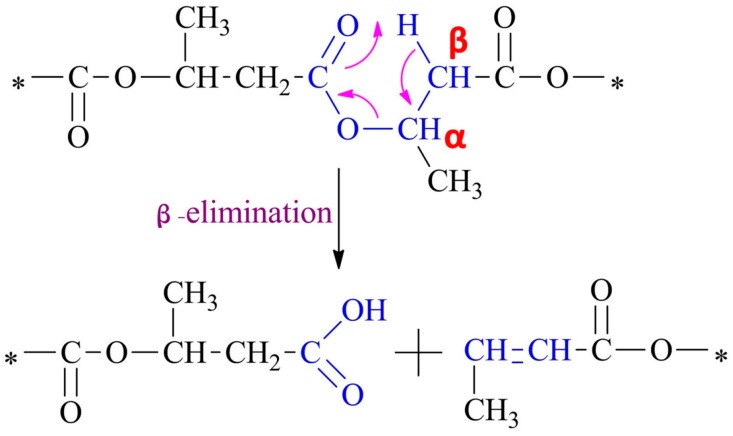
Schematic of the thermal degradation of P3HB by random chain scission reaction. Reprinted with permission from [31]. Copyright © 2008 Elsevier Ltd.

Scl-PHA can be processed into film and fiber using the melt processing method. However, the thermal stability of scl-PHA should be fully considered during the melting process. In order to process scl-PHA in a reasonable and effective way, it is necessary to study the thermal degradation mechanism and control the thermal degradation of scl-PHA. Among all the thermal degradation mechanisms of scl-PHA, the *cis*-elimination mechanism proposed by Murray et al. through analyzing the volatile components and the changes of molecular weight during the degradation process of P3HB is considered as the general pathway of scl-PHA [28]. As shown in Figure 4, a six-membered ring ester serving as a transition state is formed between the ester oxygen and neighboring β-C-H of P3HB molecular chains, which induces the thermal degradation of P3HB [29]. On the one hand, the formation of a six-membered ring is influenced by the negative inductive effect of the neighboring methylene group at the β-position to the ester oxygen [30]. The stronger the negative inductive effect of the neighboring group, the more strongly the β-C-H of the methylene group in the β-position to the ester oxygen is activated, which makes it easy to form the six-membered ring ester [31]. On the other hand, the formation of the six-membered ring ester is also affected by the electron-donating effect of a substituent group of α-position carbon atoms to the ester oxygen. The strong electron-donating effect of the substituent group makes it easy to form the six-membered ring ester [31,32].

The degradation of P3HB can be divided into two stages according to the difference of temperature. When the processing temperature was between 160 ° and 180 °C, the random chain scission of molecular chains occurred and the thermal stability of P3HB decreased significantly. Further increasing the processing temperature to 180–200 °C, a drastic decrease in molecular weight of P3HB was found due to the rapid random scission according to the β-elimination mechanism [33,34]. The sensitivity of degradation behavior on temperature led to a narrow melt processing temperature window and a limited application considering that the melting temperature of P3HB was about 180 °C. To broaden the melt processing temperature window of P3HB, a 3HV unit was introduced into the molecular chains of P3HB [32]. The melting temperature of P3HB first decreased and then increased with the increasing content of HV. When the content of HV was increased to 40 mol %, the melting temperature of PHBHV decreased from 180 °C of P3HB to 75 °C. After that, the melting temperature of PHBHV began to increase with the further increase of 3HV unit. When the content of HV was up to 95 mol %, the melting temperature of PHBHV increased to 108 °C [20,33]. Although the melt processing temperature window can be broadened to a certain extent, the cost was relatively high. Normally, the content of HV in commercial PHBHV is lower than 5 wt %.

The residual metal ions of scl-PHA can also affect the degradation process of scl-PHA [35]. It is well known that the commercialized scl-PHA usually contains a small amount of metal elements, such as Na^+^ and Ca^2+^ [36]. As a kind of Lewis acid, Ca^2+^ and Mg^2+^ can interact with carboxylic groups to seize β-H atoms that are neighboring to carbon atoms on the ester oxygen. The formation of crotonyl leads to the scission degradation of scl-PHA molecular chains. Abe et al. found that hard acid cations like Ca^2+^ and Mg^2+^ could catalyze the thermal degradation of P3HB. After the remove of residual Ca^2+^ ions using an acetic acid/chloroform solution, the weight loss temperature of P3HB increased from 240 to 275 °C [34]. Metal ions in the form of carboxylic acid salts in P3HB molecular chains can also accelerate the degradation of P3HB. A novel E1cB mechanism describing the carboxylate-induced degradation of P3HB has been proposed by Kowalczuk et al. [37]. This mechanism explains the dependence of P3HB thermal stability on the chemical structure of its end groups in the form of carboxylic acid salts. 

**Figure 5:**
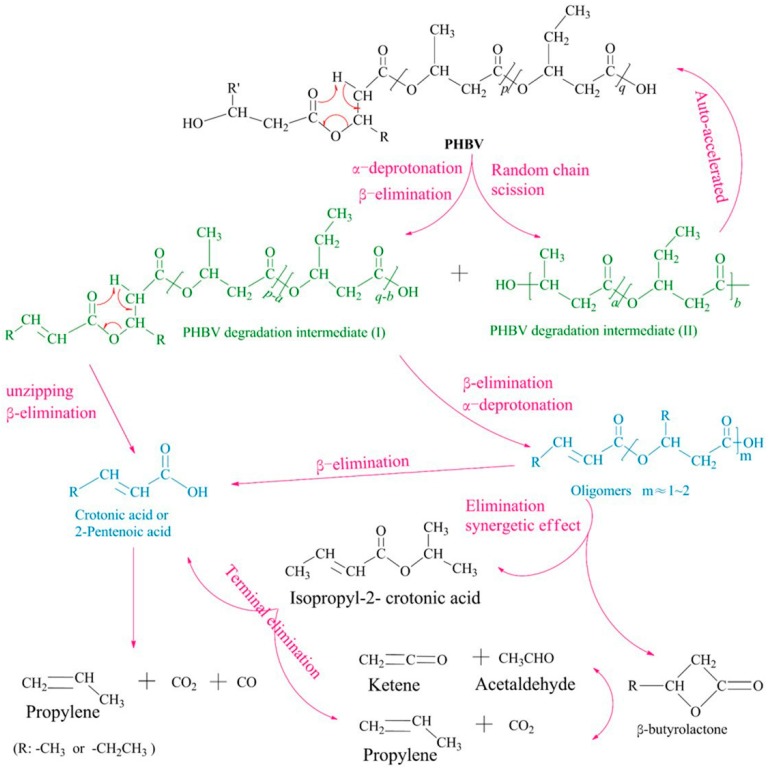
Schematic diagram of PHBHV thermal degradation. Reprinted with permission from [41]. Copyright © 2016 Elsevier Ltd.

The weight loss, structure, and molecular weight of scl-PHA during the thermal degradation process can be detected using conventional methods, including thermal gravimetric analyzer (TGA), nuclear magnetic spectrum (NMR), and gel permeation chromatography (GPC) [38,39]. In order to investigate the relationship between structure parameters and temperature or time, a series of coupling techniques are adopted to monitor the thermal process of scl-PHA, such as thermal gravimetric analysis coupled with Fourier transform infrared spectroscopy (TGA-FTIR), thermal gravimetric analysis combined with mass spectrometry (TGA-DTA/MS), or pyrolysis gas chromatography jointed with mass spectrometry (PyGC-MS) [40]. The main degradation products of PHBHV during the thermal degradation process were analyzed using the PyGC-MS technique based on β-elimination mechanism. Taking PHBHV with 30 mol % HV as an example, the main degradation products were mainly unsaturated carbonate and unsaturated ester with five carbon atoms, including propylene (6.20%), 2-butenoic acid (45.47%), 2-pentenoic acid (25.81%), propenyl-2-butenoate (11.57%), propenyl-2-pentenoate (5.39%), butyric-2-butenoate (1.93%), pentanoic-2-pentenoate (2.31%), and carbon dioxide [40]. Based on the reported research, a comprehensive description of the whole thermal degradation process and the corresponding mechanism of PHBHV are provided in Figure 5 [41].

Usually the poor thermal stability hinders the melt processing of scl-PHA. However, a series of P3HB and PHBHV oligomers were produced by Marchessault et al. from another perspective using the thermal degradation of scl-PHA [32]. Based on the work, Hakkarainen et al. developed an efficient process for chemical recycling of P3HB to functional monomers through microwave-assisted degradation in green solvents (water, methanol, and ethanol), to achieve fast hydrolysis and monomeric or oligomeric degradation products [42]. These obtained oligomers presented a well-defined structure, with a carboxylic acid end and an unsaturated end on each chain. The molecular weight of the produced oligomers was affected by the hydroxyvalerate content and could be tuned by the reaction temperature and the reaction time [32]. The oligomers of scl-PHA with low molecular weight provide the possibility for the construction of PHA-based polymers using the methods of block polymerization and graft polymerization. Furthermore, the thermal stability of P3HB with low molecular weight (LMWPHB) was significantly higher than that of neat P3HB. The addition of LMWPHB into P3HB effectively decreased the crystallinity, crystallization rate, and melting temperature of P3HB, but increased the flexibility and degradation ability of P3HB [43]. 

### 2.2. Scl-PHA-Based Composites Prepared by Physical Blending

Since no catalyst is added during the fermentation process, scl-PHA has a relatively low heterogeneous nucleation density during the crystallization process. In addition, the stereoregularity of scl-PHA is relatively high and it is easy to form a brittle scl-PHA with large-size spherulites. In order to overcome the brittleness and improve the mechanical properties of scl-PHA, physical blending becomes an economical modification method due to its low cost and simple operation. The additives incorporated into scl-PHA can be divided into nanoparticles and polymers.

#### 2.2.1. Scl-PHA/Nanocomposites

##### Influence of Nanoparticles on the Crystallization Behavior of scl-PHA

Improvement of the toughness and processability of scl-PHA can be achieved by simply introducing inorganic nanoparticles to increase the nucleation density and decrease the spherulite size [44]. In recent years, different inorganic nanoparticles have been introduced into the scl-PHA matrix by melt or solution blending. The effects of additives on the crystallization, thermal stability, mechanical properties, and biodegradability of scl-PHA have been intensively studied. For example, the role of tungsten disulphide inorganic nanotubes (INT-WS_2_) on the crystallization and melting behavior of P3HB has been discussed from a kinetic point of view [45]. It was found that the well-dispersed INT-WS_2_ played a dominant role in accelerating the crystallization rate and in reducing the activation energy barrier towards nucleation of P3HB. A significant increment of crystallization temperature of P3HB was observed when only 0.1 wt % INT-WS_2_ was incorporated into the P3HB matrix. Similar to INT-WS_2_, boron nitride (BN), talc, hydroxyapatite (HA), and zinc stearate (ZnSt) were also used as nucleation agents to modify the properties of P3HB4HB. Results suggested that BN was the most efficient nucleation agent to promote the crystallization rate of P3HB4HB, whereas the degree of crystallization was not very high. The effect of nucleation was first enhanced with the increasing content of BN and then decreased slowly when the BN content exceeded 1 wt %. On the contrary, the addition of talc increased the crystallization degree of P3HB4HB, but had little effect on the crystallization rate. Different from BN and talc, the addition of HA and ZnSt had no promoting effect on the crystallization of P3HB4HB, but decreased its crystallinity [46]. Carbon-based nanomaterial was also adopted to improve the crystallization behavior of scl-PHA. Zhu et al. investigated the effect of carbon nanotubes (CNTs) on the crystallization behavior and the nucleation mechanism of PHBHV, and found that CNTs acted as an effective heterogeneous nucleation agent, inducing an increase in crystallinity and crystal size of PHBHV. The nucleation and growth kinetics of spherulites were also discussed based on the Lauritzen–Hoffman equation. Results suggested that the temperature corresponding to the maximum spherulitic growth rate shifted to a high level with the incorporation of CNTs [47]. 

The reinforcement of scl-PHA with bio-based organic nanoparticles may hold particular interest since it would allow the development of fully biodegradable nanocomposites produced from renewable resources. The bio-based organic nanoparticles with high Young’s modulus and strength are widely used in the modification of scl-PHA [48,49], including cellulose nanocrystal (CNC) [50], chitosan nanocrystal [51], and bacterial cellulose nanowhiskers (BCNW)[52]. CNC not only owns the basic properties of cellulose, but also has a high specific surface area, a large length/diameter (L/D) ratio, and an excellent hydrophilicity [53]. Qin et al. blended CNC with PHBHV in solution and found that the incorporation of CNC increased the nucleation rate and crystallization rate of nanocomposites, leading to a narrow distribution of the PHBHV crystallite size [54]. Similar to CNC, chitosan nanocrystal also has a unique cationic structure, which is often used to enhance the properties of scl-PHAs. Gu et al. prepared PHBHV/acetylated chitin nanocrystal films using the solution-casting method and investigated the crystallization behavior of PHBHV [55]. DSC results indicated that the influence of chitin nanocrystal on the crystallization behavior of PHBHV matrix was changed from suppression to assistance after the surface modification. PHBHV/BCNW films were also prepared by Marta et al. using the same method and results showed that the incorporation of BCNW promoted the crystallization and increased the glass transition temperature of PHBHV due to the restricted polymeric chain mobility [52].

##### Effect of Nanoparticles on the Thermal Stability of scl-PHA

Based on the thermal degradation mechanism of scl-PHA, the addition of nanoparticles can improve the thermal stability of scl-PHA to a certain extent. The commonly used nanoparticles include titanium dioxide (TiO_2_) [56], LDH [57], and clay [58,59,60]. Buzarovska et al. found that the decomposition temperature of PHBHV increased from 236 to 253 °C when 1 wt % TiO_2_ was incorporated, and the decomposition temperature further increased to 265 °C when the content of TiO_2_ was up to 10 wt % [56]. The thermal degradation behavior of P3HB/poly(ethylene glycol) phosphonates (PEOPAs)-modified layered double hydroxide (PMLDH) nanocomposites was also investigated. The calculated *E_a_* value of P3HB/PMLDH nanocomposites was increased with the increasing content of PMLDH, which could be attributed to the incorporation of more PMLDH loading to P3HB, inducing a decrease in the degradation rate and an increase in the residual weight [57]. Bruzaud et al. prepared PHBHV/organically modified montmorillonite by the solution intercalation method and found that the thermal stability was greatly enhanced by the addition of only a small percentage of organoclay [58]. For example, the temperature corresponding to 50% degradation of PHBHV-based nanocomposite increased from 270 to 300 °C with the addition of 5 wt % organoclay, meaning that the thermal decomposition process of PHBHV/organoclay composite took more time to start in the presence of a small percentage of organoclay. For the organic nanoparticles, several studies showed that CNC was able to improve the thermal stability of PHAs [54], whereas a slight decrease in the thermal degradation was observed in other cases [61,62]. Similar to CNC, the degradation onset and maximum degradation temperature of PHBHV were slightly shifted from 239.4 to 245.0 °C and 287.0 to 290.1 °C, respectively, when 1 wt % BCNW was incorporated. This phenomenon was caused by the formation of a strong network held by hydrogen bonds between the matrix and the nanofiller. However, further increases in nanofiller loading resulted in decreased thermal stability of composites [52].

##### Mechanical Properties of scl-PHA Improved by Nanoparticles

In addition to accelerating nucleation and improving the thermal stability of scl-PHA, nanoparticles can also improve the mechanical properties of scl-PHA. Park et al. reported the PHBHV nanocomposite reinforced by graphene using the solution blending method. Samples with 6 wt % graphene presented substantially high storage modulus, which was attributed to the increased immobilization of PHBHV chains onto the surface of graphene [63]. Mualla et al. prepared PHBHV bio-composites with incorporating untreated hydroxyapatite (HAP) and silane-treated HAP (sHAP) nanoparticles using melt extrusion processing. Results showed that modified HAP could be uniformly dispersed in PHBHV matrix and produced the toughness effect at an appropriate addition. The Young’s modulus and tensile strength of PHBHV composite were increased by 15% and 48% with the addition of 5 wt % sHAP, respectively [64]. Similar to HAP, the presence of cellulose nanowhiskers (CNW) promoted a considerable chain orientation in the same direction of the applied load and greatly enhanced the elongation-at-break of PHBHV through the slippage of CNW and the oriented molecules during the plastic deformation process [65]. By incorporating 5 wt % CNW, the PHBHV/ CNW film showed 77% improvement in Young’s modulus, 35.5% increase in tensile strength, and 41% increase in storage modulus, indicating that CNW was an effective reinforcing agent for PHBHV [62]. To further improve the interfacial adhesion and mechanical performance of scl-PHA, modified CNC (mCNC) with good compatibility was adopted. The storage modulus of P3HB4HB/mCNC nanocomposites prepared by solution casting method was enhanced by 47% when incorporated with 3 wt % mCNC, which was ascribed to the uniform dispersion of mCNC and the strong interfacial adhesion between mCNC and the matrix. [66].

#### 2.2.2. Scl-PHA/Polymer Composites

##### Influence of Polymers on the Crystallization Behavior of scl-PHA

Blending scl-PHA with other polymers is a practical and economical approach to tailor its crystallization behavior [67]. The blended polymers can be divided into non-biodegradable and degradable polymers. As for non-biodegradable polymers, the crystallization behavior of incompatible crystalline/amorphous blends (such as PHBHV/polystyrene (PS) and PHBHV/polymethyl methacrylate (PMMA)) under different ratios and different crystallization temperatures has been intensively investigated [68]. Results suggested that the dispersion state and blend ratio of amorphous content had a decisive effect on the formation of PHBHV ring-banded spherulites. Similar results were also found in PHBHV/thermoplastic polyurethane (TPU) composites [67]. The addition of amorphous TPU decreased the crystallinity of PHBHV and damaged the integrity of PHBHV spherulites; however, banded spherulites of PHBHV still existed. The crystallization kinetics of P3HB and P3HB/polymer vinyl acetate (PVAc) composites was investigated using broadband dielectric technique over a wide range of frequencies (10^−2^–10^5^ Hz). The dielectric intensity of the amorphous segments, which was proportional to the volume of the mobile amorphous phase, decreased exponentially with the increase of the crystallization time, while the dielectric strength of the rigid amorphous segments, which is related to the percentage of crystallinity in the composite, increased dramatically with the increase of the crystallization time [69].

Blending scl-PHA with degradable polymers attracts even more interest, considering that non-degradable polymers may pose environmental problems in the future. Among all the degradable polymers, polycaprolactone (PCL) [70], poly(lactic acid) (PLA), poly(butylene succinate) (PBS), lignin [71], and plant fibers [72] were used to control the crystallization process of scl-PHA and to prepare fully degradable composites [73]. Müller et al. reported the influence of PCL with different molecular weights on the properties of P3HB. PCL with high molecular weight was immiscible with P3HB despite the proportion of composite, leading to a separated crystallization phenomenon. By decreasing the molecular weight of PCL, a partially miscible phenomenon was observed in the P3HB/PCL composite [74]. Ozaki et al. investigated the miscibility of P3HB/PHBHHx/PLLA composites with different ratios of P3HB, PHBHHx, and PLLA. Results showed that the crystal lattice parameters of *a* and *b* did not change with the changing of component ratios, suggesting that the crystal structures of these polymers did not depend on the second component [75]. The addition of PBS improved the thermal stability and crystallization property of P3HB4HB. A better compatibility between P3HB4HB and PBS phases and a mutual promotion on the crystallization property were found when the content of PBS was less than 30 wt % [76]. The effects of lignin on the crystallization process of PHBHV could be classified in two opposite ways, namely the enhanced effect of nucleation and the hindered effect of spherulites’ growth [71]. Unlike the abovementioned polymers, the addition of kenaf fibers had little effect on the crystallization kinetics of PHBHV, as analyzed by Buzarovska et al. using Avrami and Mo’s models [77].

##### Effect of Polymers on the Thermal Stability of scl-PHA

Similar to nanoparticles, some bio-based or petroleum-based polymers have also been used to improve the thermal stability of scl-PHA. Roa et al. improved the thermal stability of P3HB through the molecular interaction between the terminal hydroxyl groups from poly(propylene glycol) with low molecular weight and the carbonyl groups from P3HB [78]. Blends of P3HB with PHO containing 98 mol % 3HV and 2 mol % 3HHx were prepared by melt compounding. Results showed that the degradation onset temperature of P3HB gradually increased with the addition of PHO, and the shift in temperature was as high as 20 °C for the blends containing 30 wt % PHO [79]. Idris et al. investigated the effect of PLA on the thermal stability of PHBHV and found that the decomposition temperature of PHBHV increased with the increasing content of PLA due to the higher thermal stability of PLA. Besides, the decomposition behavior of PHBHV changed from one-step degradation to two-step degradation [80]. A similar phenomenon was also found in PHBHV/epoxidized broccoli oil (EBO) blends [81].

##### Mechanical Properties of scl-PHA Enhanced by Polymers

**Figure 6:**
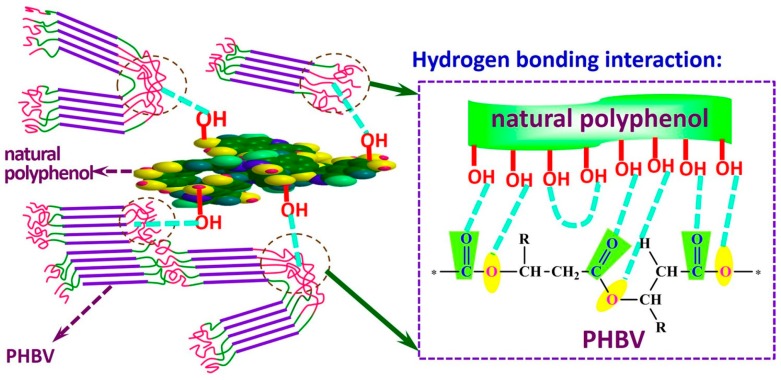
Schematic diagram of the formation of hydrogen bonding interaction between PHBHV and natural polyphenol.

Polymers with excellent melt processability are also used to improve the physical properties and reduce the cost of scl-PHA-based composites [82]. It has been reported that the miscibility between scl-PHA and other polymers is far from satisfactory. Therefore, compatibilizers are always used to improve the compatibility between the phases of scl-PHA and other polymers. For example, 5 wt % oxidized polyethylene wax (OPW) was added into the blends of P3HB and low-density polyethylene (LDPE) to improve the compatibility of the composite, and results showed that the tensile strength and Young’s modulus of obtained composite were increased by the use of OPW [83]. Similar to petroleum-based polymer, PLA [84], wood flour [85], and plant polyphenols were used to improve the mechanical properties of scl-PHA without counteracting its degradable property [86]. Normally, the incorporated polymers must be thoroughly dried to avoid the degradation of scl-PHA caused by moisture during the melt processing. Otherwise, the molecular weight of scl-PHA will decrease due to the hydrolysis reaction induced by residual moisture [87,88]. Ma et al. prepared PHBHV/ PLA composites by melt blending and found that the ductility and toughness of PHBHV/ PLA composites improved significantly when the content of PHBHV was 10–30 wt %. As they mentioned, the fibrillation, partial interfacial debonding, PHBHV domain cavitation, and matrix yielding were involved in the toughening mechanism of the fully bio-based blends [89]. Gregorova et al. reported P3HB films reinforced with inexpensive wood flour using the melt pressing method and studied the surface modification of beech wood flour on the mechanical properties of P3HB. Results showed that the addition of 20% SA-WF (stearic acid treated wood flour) increased the tensile strength and Young’s modulus from 36.4 MPa and 2990 MPa of P3HB film to 39.6 MPa and 3420 MPa of P3HB/SA-WF film, respectively. In order to avoid the thermal degradation and brittle transition of PHBHV, natural plant polyphenols, such as tea polyphenols (TP) [90] and tannic acids (TA) [91], were also selected to improve the mechanical properties of PHBHV. The crystallization behavior as well as the mechanical properties of PHBHV could be regulated by the hydrogen bonding formed between plant polyphenols and PHBHV, as shown in Figure 6. The elongation-at-break of PHBHV/TP composite films improved from 1.5% to 36.5%, while the tensile strength decreased from 14.0 to 6.6 MPa when 20% TP was incorporated. Based on the work, PHBHV/TA films were prepared by the solvent casting method. Results showed that the elongation-at-break and tensile strength of PHBHV/TA films increased to 14% and 23.6 MPa when the percentage of added TA reached 10%.

### 2.3. Chemical Structure Design of scl-PHA

#### 2.3.1. Influence of Chemical Structure Design on the Crystallization Behavior of scl-PHA

To solve the disadvantages of scl-PHA mentioned above, many studies have focused on chemical modification in an effort to regulate the stereoregularity of scl-PHA. The chemical structure of scl-PHA can be tailored by controlling the interaction between the molecular chains of scl-PHA and the incorporated components using grafting, block copolymerization, or crosslinking methods [92].

##### Graft Modification of scl-PHA

By incorporating other components into scl-PHA chains with the method of grafting, the compatibility and crystallization behavior of scl-PHA can be effectively controlled through the formation of chemical bonds and non-covalent bonds between the two components. Gu et al. synthesized PHBHV-*g*-chitin by grafting PHBHV molecular chains onto a chitin backbone via chlorination. Although the surface modification reduced the number of hydroxyl groups of chitin, the crystallization of PHBHV was still limited due to the formation of hydrogen bonds between the carbonyls from PHBHV and the residual hydroxyls from chitin [93]. Similar to this work, the influence of the side-chain length and grafting density on the crystallization behavior of cellulose (EC)-*g*-PHBHV, synthesized through the homogeneous acylation reaction between EC a backbone and telechelic OH-terminated PHBHV oligomer as side chains by using 1,6-hexamethylene diisocyanate (HDI) as a coupling agent, has also been investigated [94]. Results showed that the crystallization behavior can be modulated through controlling the lengths and grafting densities of PHBHV side chains. The degree of crystallinity of EC-*g*-PHBHV with a HDI/PHBHV fraction of 1.8 decreased from 58.1% to 39.1% compared with that of PHBHV. 

##### scl-PHA-Based Block Copolymers

Since the molecular weight of scl-PHA is relatively high, it is hard to design a scl-PHA-based structure with excellent properties. Tailoring the molecules of scl-PHA into oligomers with different molecular weights and narrow MWD becomes a convenient method to design the chemical structure of scl-PHA-based polymers. Telechelic hydroxylated scl-PHA (scl-PHA-diols) with various molecular weights were obtained by the attack of hydroxyl groups of ethylene glycol to ester groups of scl-PHA [95]. PHBHV-*b*-PEG [96] and P3HB-based polyurethane block copolymer (PEU) [97,98] were synthesized by using the coupling reaction with scl-PHA-diols as hard segments and PEG as soft segments. It has been found that the block copolymers had higher thermal stability but lower melting point and crystallinity compared with those of scl-PHA-diols. The crystallization behavior of PHBHV and PEG in double crystalline block copolymers was also investigated. Results suggested that the spherulites of PEG block and PHBHV block in the PHBHV-*b*-PEG copolymer were crystallized individually on the same site, leading to the formation of concentric spherulites [99]. Zhu et al. studied the influence of molecular structure on the crystallization behavior and thermal stability of PHBHV in an effort to obtain a copolymer with high thermal stability and mechanical properties. A series of crystalline/amorphous copolymers were designed by chemical block copolymerization, including PMMA-*b*-PHBHV [100] and PS-*b*-PHBHV [101] triblock copolymers. They found that the cold crystallization temperature of both PMMA-*b*-PHBHV and PS-*b*-PHBHV were shifted to a higher temperature with the increasing content of amorphous component, and for PMMA-*b*-PHBHV the increased value was up to 60 °C. What is more, scl-PHA-based block-polymers (*b*-PHA) can also be synthesized using biological technology; the crystallization behavior and mechanical properties of scl-PHA-based copolymers depended on the structure and content of incorporated components [102,103,104,105].

##### Crosslinking Modification of scl-PHA

**Figure 7:**
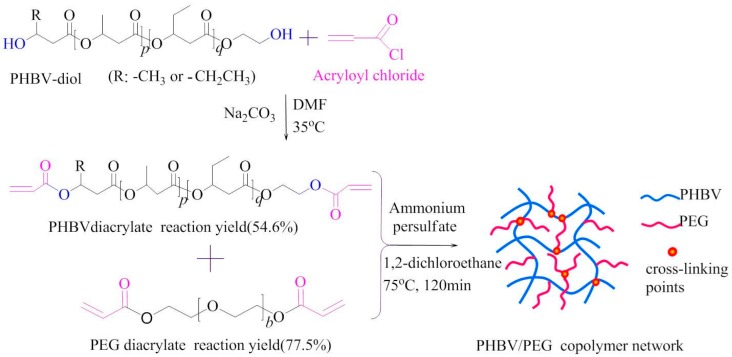
Synthesis of PHBHV/PEG copolymer network. Reprinted with permission from [107]. Copyright © 2013 Science China Press and Springer-Verlag Berlin Heidelberg.

Partially cross-linked scl-PHA exhibits an improved melt strength characteristic without damaging its biodegradability, which can be induced by chemical, radiation, light, or thermal treatment. The processing performance of scl-PHA can be effectively improved by the partially cross-linked structure and the enhanced chain entanglement. A partially cross-linked PHBHV/PBS composite was prepared by melt blending and the overall crystallization rates of both PHBHV and PBS in their blends were enhanced considerably. In addition, the nuclei density of PHBHV was also increased while the spherulitic morphology did not change too much [106]. PHBHV/PEG copolymer networks were also synthesized by using the free-radical solution polymerization method, as shown in Figure 7 [107]. The macromolecular network hindered the growth of crystalline segments of PEG and PHBHV, and a complex spherulite morphology with an initial PHBHV pattern and later PEG pattern was observed in the partially cross-linked PHBHV/PEG copolymer. This complexity was induced by the crystallization of PHBHV followed by the crystallization of PEG, which was an interplay of the nucleation rate and growth rate. Dong et al. prepared a series of branched/crosslinked P3HB4HB copolymers [108] and PLA/P3HB4HB composites with high compatibility [109] by adding small amounts of dicumyl peroxide (DCP) and triallylisocyanurate (TAIC) into a P3HB4HB matrix. The cold crystallization ability of the copolymer was increased by the branching of molecular chains while the melting temperature was decreased. The branches of molecular chains increased the space between molecular chains after the branching reaction, which provided the space for the motion of molecular chains during the heating process. As a result, the cold crystallization ability of P3HB4HB was enhanced. On the contrary, the disordered branches led to a defective crystal and a lower *T*_m_ [108].

#### 2.3.2. Effect of Chemical Structure Design on the Thermal Properties of scl-PHA

**Figure 8:**
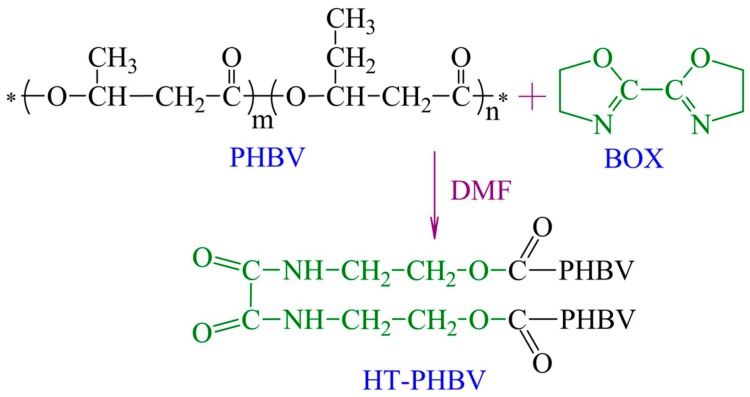
Reaction between PHBHV and BOX. Reprinted with permission from [31]. Copyright © 2008 Elsevier Ltd.

Chemical structure design methods are used to change the chemical bonding environment of a six-membered ring ester in an effort to improve the thermal stability of scl-PHA [37]. Ma et al. synthesized P3HB-*g*-MA (maleic anhydride) with the assistance of styrene using the melt free-radical grafting method. The graft ratio of MA on the P3HB macromolecule chains increased from 0.2 to 0.9 wt % and the degradation temperature (*T_d_*_-5%_) of P3HB increased by 35 °C [110]. The thermal stability of PS-*b*-PHBHV triblock copolymers was analyzed by TGA and results showed that the *T_d_*_-5%_ and *T*_max_ of triblock copolymers increased by 30 and 40 °C compared with those of PHBHV, respectively [101]. Park et al. found that the thermal stability of P3HB was improved by the crosslinking reaction between the epoxy group and terminal carboxyl group in polyglycidyl methacrylate (PGMA) [111]. In addition, chain extension could also enhance the thermal stability of PHBHV by changing the chemical environment of ester linkages to reduce the formation of a six-membered ring ester during the thermal degradation process, as shown in Figure 8. Zhu et al. used 2,2-bis (2-oxazoline) (BOX) as a chain extender and found that the *T*_5%_, *T*_0_, *T*_max_, and *T*_f_ of hydroxyl-terminated PHBHV (HT-PHBV) increased by 30.6, 24.2, 19.4, and 19.1 °C, respectively [31].

#### 2.3.3. Mechanical Properties of scl-PHA Improved by Chemical Structure Design

Chemical modification is usually needed to conduct with physical blending in order to obtain a satisfactory scl-PHA with excellent properties. The effect of partial crosslinking initiated by dicumyl peroxide (DCP) on the performance of PHBHV has been investigated. Dong et al. found that the elongation of PHBHV increased from 4% to 11% when 1 wt % DCP was added [112]. They also found that the tensile/flexural strength and impact toughness of P3HB/Poly(d,l-lactic acid) (PDLLA) blends were improved after the partially crosslinking regardless of the composition of the blends, which was ascribed to the improvement of the compatibility between P3HB and PDLLA [113]. The mechanical properties of PEU synthesized using the block copolymerization were also investigated. All of the block copolymers had lower Young’s modulus and stress-at-break at 177 to 616 MPa and 9 to 18 MPa, respectively, as well as higher strain-at-break at 12% to 1090% as compared with those of pristine PHB, which were 1143, 26 MPa, and 2%, respectively [108]. The improvement in ductility was attributed to the reduction of P3HB crystallinity, the presence of urethane bonds, and the plasticization effect of soft PEG segments. 

### 2.4. Processing of scl-PHA 

#### 2.4.1. Influence of Processing Condition on the Crystallization Behavior of scl-PHA

As a kind of linear bio-based polyester, scl-PHA owns a high stereoregularity and a low crystallization rate, leading to a large and visible banded spherulite. The crystal structure and crystallization behavior of scl-PHA are not only related to its structure, but also depend on the processing conditions. Similar with the chemical structure, processing conditions also have a great influence on the aggregation structure of scl-PHA, which determines the final performance of scl-PHA products. Many studies have focused on processing modifications in an effort to regulate the crystal structure and crystallization behavior of scl-PHA [114]. For a given polymer, the crystallization rate mainly depends on the processing temperature. An optimum balance can be reached between chain mobility and lamellae growth at an intermediate temperature between *T*_m_ and *T*_g_. Barham et al. investigated the long period and unit cell dimension of PHBHV with 0–27 mol % HV content crystallized at 23, 52, and 81 °C. Results suggested that the amount of co-crystallization of HV in the crystal structure of P3HB decreased with the increase of crystallization temperature, and the amount of HV in the crystal formed at 81 °C was reduced to two-thirds of that formed at room temperature [115]. The crystal structure and spherulite morphology of P3HB crystallized from melt film and *N*,*N*-dimethyl formamide solution were also investigated, as well as the formation mechanism of the banded spherulite under different crystallization conditions [116]. At the beginning of the crystallization of P3HB from melt, the spherulite morphology was determined by the crystallization rate of crystalline segments and the diffusion rate of molecules on the surface of crystals, while the spherulite morphology of P3HB crystallized from solution was mainly affected by the twisted alignment of lamellas. The crystallization behavior of scl-PHA has also been reported to be related to the thickness of the samples. Wang et al. found that the banded spherulite, which was mainly determined by the ratio between crystallization rate and diffusion rate, was easy to form by decreasing the thickness of P3HB [117].

**Figure 9:**
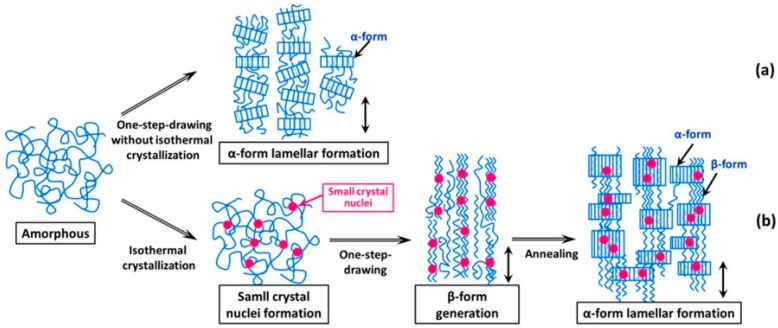
Mechanism for generating the planar zigzag conformation (β-form) in high-strength PHBHV fibers by different drawing methods: (**a**) one-step-drawing without isothermal crystallization; (**b**) one-step-drawing after isothermal crystallization. Reprinted with permission from [121]. Copyright 2006 American Chemical Society.

Besides the mentioned factors, the crystallization behavior of scl-PHA can also be effectively controlled by the application of external fields during the process [118,119]. For example, cold-drawing and two-step-drawing methods were used to increase the orientation of scl-PHA molecular chains and generate a planar zigzag conformation. By using these special drawing techniques, Iwata et al. successfully prepared P3HB [120] and PHBHV [121] fibers and films with high mechanical properties. A new formation mechanism of β-form crystals appearing in one-step drawn PHBHV fibers after isothermal crystallization can be described as follows: the α-form lamellar crystals were produced by cold-drawing and then the β-form with planar zigzag conformation was developed during the stretching in the second dimension of the constrained amorphous chains between α-form crystals, as depicted in Figure 9 [121].

#### 2.4.2. Effect of Processing Conditions on the Mechanical Properties of scl-PHA

**Figure 10:**
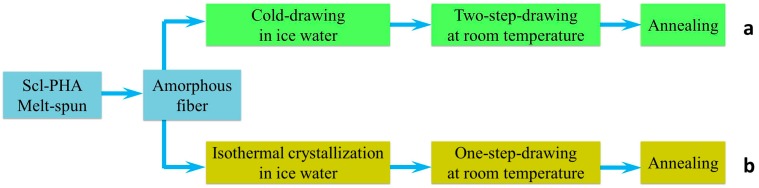
Preparation methods for PHA fibers: (**a**) One-step drawing without isothermal crystallization and (**b**) One-step drawing after isothermal crystallization. Reprinted with permission from [121]. Copyright 2006 American Chemical Society.

The processing conditions have a great effect on the mechanical properties of scl-PHA. Scl-PHA with excellent properties can be obtained under different processing conditions due to the formation of a special aggregation structure. Table 2 shows the mechanical properties of different scl-PHAs obtained under different treatment conditions. After special treatment, highly orientated crystals can be formed to enhance the mechanical properties of scl-PHA. Gordeyev et al. used the three-step stretch method and obtained P3HB gel-spun fibers with a tensile strength of 360 MPa and a Young’s modulus of 5.6 GPa [122]. The enhancement of the mechanical properties of scl-PHA was not only caused by the orientation of molecular chains but also by the formation of a planar zigzag conformation. Iwata’s group established two kinds of drawing techniques (Figure 10) and obtained P3HB fibers with high tensile strength. As shown in Figure 10a, the melt-spun scl-PHA fibers were quickly treated in ice water and stretched at a temperature near the glass transition temperature. The orientated amorphous fibers were further stretched at room temperature and then annealed to fix the extended polymer chains [123]. P3HB fiber with a tensile strength of 1320 MPa, a Young’s modulus of 18.1 GPa, and an elongation of 35% was obtained using this method. Figure 10b presents another method for preparing scl-PHA fibers. The melt-spun fibers were also placed in the ice water bath for isothermal crystallization, and crystals with small size were formed during the slow crystallization process. After that, the scl-PHA fibers were obtained by using a one-step-drawing method at room temperature. The tensile strength and elongation-at-break of obtained PHBHV fibers could reach up to 1065 MPa and 40%, respectively [121]. 

The mechanical properties of scl-PHA-based composites can also be enhanced by improving the orientation degree of nanoparticles in composites with the help of an external field. Wolcott et al. prepared the unidirectionally aligned CNW in a PHBHV matrix by using an external electric field. It was found that the arrangement of CNW in a PHBHV matrix was largely affected by the concentration of CNW. When the concentration of CNW was higher than 4 wt %, the movement of CNW would be limited by the high viscosity of the PHBHV/CNW suspension [125].

## 3. Potential Applications of scl-PHA

PHA is the only bio-based polyester family completely synthesized by biological means, and scl-PHA shows a significant potential for future applications in the packaging, fiber, and biomedical fields. The main reason is that scl-PHA has excellent biological compatibility, a long absorbable period, and similar properties to general petroleum-based polymers. Moreover, the large-scale industrial production of scl-PHA can be carried out in a green way without complex equipment, according to a United States Patent (US 7582456 B2). 

### 3.1. Scl-PHA as Packaging Material

**Figure 11:**
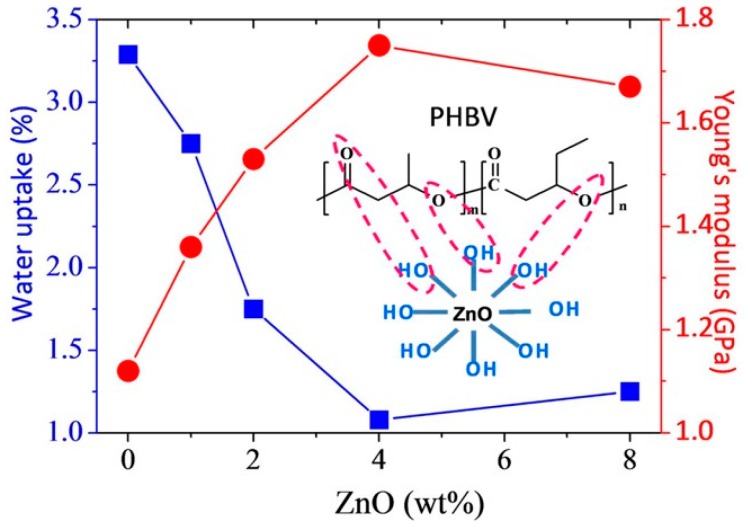
Young’s modulus and water uptake of PHBHV/ZnO film. Reprinted with permission from [132]. Copyright 2014 American Chemical Society.

Biodegradable packaging materials have potential as replacements for petroleum-based materials to reduce environmental pollution. Biodegradable packaging materials can be degraded into carbon dioxide and water by microorganisms via metabolism in the natural condition with suitable temperature, humidity, and oxygen [126,127]. As the only biodegradable polymer that comes entirely from nature, scl-PHA can be processed into bottles, bags, and films using injection molding, blowing, and pressing methods due to its good properties of biodegradability and oxygen permeability resistance [128,129]. Coussy et al. investigated the sorption behavior, mechanical properties, and barrier properties of PHBHV film under realistic conditions of storage and food contact. It was found that PHBHV film was very stable in all the food liquid simulants tested (water, acetic acid 3% (*w*/*v*), ethanol 20% (*v*/*v*), iso-octane and olive oil) [130]. Kovalcik et al. prepared PHBHV/Kraft lignin films using a thermo-hydraulic press, which can be applied for packaging of various easily perishable goods [131]. A reduction of permeability for oxygen by 77% and for carbon dioxide by 91% was observed compared to neat PHBHV when 1 wt % lignin was incorporated. Diez-Vicente et al. dispersed ZnO into scl-PHA with the method of solution spin coating and obtained a composite film with antibacterial properties, which could be applied in the food packaging field. Without the use of a coupling agent, nano-ZnO achieved a uniform dispersion in a PHBHV matrix, and also proved effective in the nucleation of PHBHV. With the increased addition of nano-ZnO, the thermal property, rigidity, strength, and tenacity of PHBHV film increased significantly. As shown in Figure 11, when the content of nano-ZnO was up to 4 wt %, the Young’s modulus of PHBHV film reached a maximum while the water absorption reduced a minimum. This suggests that interactions were formed between hydroxyl groups from the surface of ZnO and C=O groups from PHBHV molecular chains, which improved the mechanical properties and gas barrier properties of the PHBHV film [132].

However, the brittleness still restricts the further application of scl-PHA as a packaging material. In order to solve this problem, Ariffin et al. incorporated PHBHV into petroleum-based polyethylene (PE) and evaluated the recyclability of the PE/PHBHV composite. It was found that the oxygen permeability of PE/PHBHV blend films containing up to 30 wt % PHBHV was reduced by 19%–25%, and their water vapor transmission rate increased compared to those of neat PE. Furthermore, PHBHV in composite could be completely degraded into monomers and isolated from PE under 310 °C without polluting PE, and the PE component could be recycled and reused by other methods [133]. Similar to this work, bio-degradable and optically transparent films were prepared by blending PLA with P3HB using the melt processing method. P3HB and acetyl tributyl citrate (ATBC) were used to improve the crystallinity of PLA and the processability as well as the flexibility of the film. In addition, the thermal stability of PLA/P3HB film could be improved by the addition of CNC. The interaction between ATBC and CNC improved the dispersion of CNC, and further increased the intermolecular interaction between PLA and PHBHV chains [134].

### 3.2. Scl-PHA as a Fiber Material

Scl-PHA fibers can be obtained using melt spinning, gel spinning, or electrospinning methods. In terms of melt spinning, Zhu et al. made great efforts to improve the melt spinnability of PHBHV. It was discovered that the melt spinning temperature of PHBHV was around 180–190 °C after exploring the relationships between viscosity, shear rate, and temperature. However, the conventional melt spinning method was not suitable for the spinning of neat PHBHV. PHBHV fibers with a strength of 1.85 cN/dtex and an elongation of 47.9% could be obtained after stretching through a high-speed nozzle [135]. After the formation of scl-PHA fibers, a transition process from viscoelasticity to brittleness could be observed with the increase of storage time, which restricted their further application. To solve the problem, Chen et al. blended PLA with PHBHV and obtained PLA/PHBHV fibers via the conventional melt-spinning and hot-drawing processes [136]. The tensile strength of PLA/PHBHV (70/30) fibers obtained under a take-up speed of 2500 m/min and a draw ratio of 1.6 was above 2.0 cN/dtex, which pointed to a potential application in the textile field. Hufenus et al. combined PHBHV with PLA in a core/sheath configuration and introduced a new spin pack concept [137]. The obtained fibers showed an ultimate tensile stress of up to 0.34 GPa and an *E*-modulus of up to 7.1 GPa. To further improve the mechanical properties of scl-PHA fibers, drawing and heat setting processes were applied after the formation of P3HB and PHBHV fibers [138]. Besides the melt spinning method, scl-PHA fibers can also be prepared using the gel spinning method, which contains the processes of extrusion, hot drawing, and annealing. Gordeyev et al. prepared the gel-spun P3HB fibers with a tensile strength of 360 MPa and a Young’s modulus of 5.6 GPa [122]. Different to melt and gel spinning, the electrospinning method is often used to prepare scl-PHA nano-fibers with special morphology [139], such as self-bundling yarn [140], coral-like structure [141], and beaded structure [142], which are a useful prospect in the areas of tissue engineering, filtration, and sensors. Electrospun scl-PHA fibers also have porous surface structure and microstructure, which provides the possibility for hydrophilic or hydrophobic control and for the adhesion regeneration of cells. For example, the contact angle of PHBHV electrospun films could be controlled from 75.9° to 158° [143]. Moreover, the surface structure of nano-fibers can be further adjusted to control the interfacial parameters using plasma treatment technology [142].

### 3.3. Scl-PHA as Biomaterial

**Figure 12:**
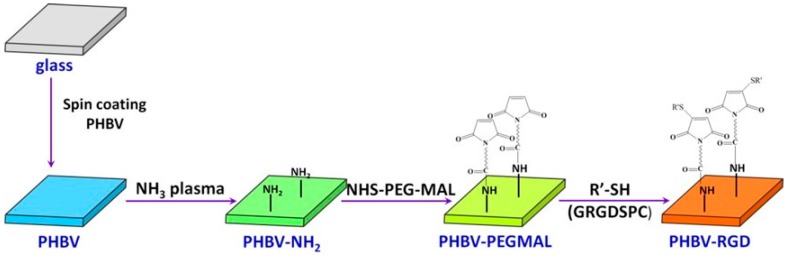
Preparation and surface modification of PHBHV film. Reprinted with permission from [151]. Copyright 2011 American Chemical Society.

Scl-PHA is conducive to the adhesion and proliferation of cells due to its excellent biocompatibility and biodegradability, which makes it a hot topic in the areas of tissue engineering and biomedical field [144]. In terms of the applications in tissue engineering, Stanzl-Tschegg et al. investigated the adhesive strength of bone-implant interface and in vivo degradation of P3HB composites [145]. Results showed that P3HB composites with ZrO_2_ and a high percentage of Herafill^®^ (30%) (which is a composite made of calcium sulfate (CaSO_4_), calcium carbonate (CaCO_3_) and glycerol tripalmitate) showed the highest values of bone accumulation around the implant and no significant degradation of the implants was found after 36 weeks in vivo. However, improvement of the mechanical properties of the studied P3HB composites was necessary in order to obtain an appropriate load-bearing material. Similar to physical blending, methods such as surface modification [146] and chemical structure design [147,148] are adopted to improve the hydrophobicity of scl-PHA films and the recognition site of cells. Grondahl et al. introduced carboxylic acid groups [149] and amino groups [150] onto the surface of PHBHV using the radiation grafting method and ammonia plasma treatment to facilitate the adhesion of biological groups. Based on the work, Huang et al. activated the surface of amino modified PHBHV film after a continuous reaction with cross-linking agents containing polyethylene glycol (PEG) and amino acid (RGD), as described in Figure 12 [151]. It was found that the introduced PEG segments significantly improved the biological compatibility and decreased the non-specific absorption and the risk of the formation of blood clots. PUHO scaffold was also prepared by coupling P3HB4HB with poly(3-hydroxyhexanoate-*co*-3-hydroxyoctanoate) (P3HH3HO) with the help of 1,6-hexamethylene diisocyanate (HDI) [147,148]. Results of lactate dehydrogenase (LDH) and platelet adhesion tests showed that the platelet adhesion property of PUHO scaffold was higher than that of commercial PLA material. 

For the application of scl-PHA in the medical field, Koller et al. synthesized PHBHV and P(3HB-*co*-21.8%-3HV-*co*-5.1%-4HB) by a biological method using *Haloferax mediterranei.* Both PHAs had low melting points and narrow molecular mass distributions, so could be used as potential candidates for the application in medical and pharmaceutical fields [152]. Scl-PHA microsphere shows an advantage in drug delivery and controlled release material due to its slow degradation rate. The degraded scl-PHA microsphere in the body provides the space for tissue growth, and the degraded products would not produce acid accumulation, which can cause tissue inflammation [153,154]. Chang et al. prepared a PHBHV/wollastonite composite microsphere and incorporated gentamicin into the microsphere [155]. They found that the release behavior of gentamicin from the composite microspheres in deionized water was similar to that from pure PHBHV. However, the release rates of microspheres in a simulated body fluid (SBF) and phosphate buffer solution (PBS) were much slower than those of pure PHBHV. The release time of 90% gentamicin for PHBHV/wollastonite microsphere was 22 days, while that for pure PHBHV microsphere was only eight days. The mechanical properties and processability of slow-released microsphere can be enhanced by the addition of chitosan (CTS) [156]. Wang et al. synthesized the drug-releasing microspheres of P3HB/CTS by single and double emulsion methods and found that the size of microspheres could be controlled in the range of 800 nm–2 μm [157]. 

### 3.4. Other Potential Applications of scl-PHA

#### 3.4.1. Scl-PHA as a Source of Biofuel

The application of scl-PHA as a source of biofuel is promising due to the fact that the low-cost scl-PHA used in this process can be obtained from activated sludge or food industry waste [158,159]. The role of scl-PHA-based materials as biofuels was first proposed by Chen et al. in 2009 [160]. P3HB methyl ester (3HBME) with a purity of 97% was synthesized by the esterification of P3HB via acid-catalyzed hydrolysis. Results showed that the combustion heats of 3HBME and ethanol were 20 and 27 kJ/g, respectively, while that of their blend with 10% 3HBME content was increased to 30 kJ/g. The production cost of PHA-based biofuels was also roughly estimated, which should be around $1200 per ton. In their subsequent work, they blended 3HBME with 97# gasoline in volume ratios of 5%, 8.5%, 10%, 15%, and 20% and found that 3HBME had similar or better properties as a fuel additive compared with ethanol in terms of oxygen content, dynamic viscosity, flash point, and boiling point [161]. Similar work has also been reported by Chang et al. in an effort to produce biofuel *n-*butanol from glucose using an adapted P3HB synthesis pathway together with other metabolic pathways [162].

#### 3.4.2. Scl-PHA as a Precursor of Carbon Material

As a kind of polymer with plenty of alkyls and hydroxy fatty acids, scl-PHA can introduce an additional oxygen element into a carbon precursor. Zhu et al. synthesized a spindle-like hierarchical carbon structure of submicron dimension by the pyrolysis of a PHA/ferrocene/chloroform precursor [163]. The electrochemical performance of the obtained carbon material showed a specific capacitance of 188 F/g with excellent stability over 10,000 cycles. In addition, scl-PHA can also be used to improve the melt spinnability of Kraft lignin and prepare continuously spooled lignin/scl-PHA fibers, which could be converted to low-cost carbon fibers. Since the glass transition temperature of Kraft lignin is relatively high, it is difficult to obtain continuously spooled lignin fibers due to the fact that lignin melt will become brittle before being spooled. The incorporation of polymers with good melt spinnability can improve the spinnabiliy of lignin [164], especially polymers that can maintain the molten state for a long time during the melt spinning process. This special requirement seems to be tailored for scl-PHA with a slow crystallization rate and a low glass transition temperature. We found that the addition of 5% PHBHV could effectively improve the melt spinnability of lignin and the obtained lignin/PHBHV composite fibers were successfully converted to carbon fibers. Related work is ongoing and will be published in the near future.

## 4. Conclusions

**Figure 13:**
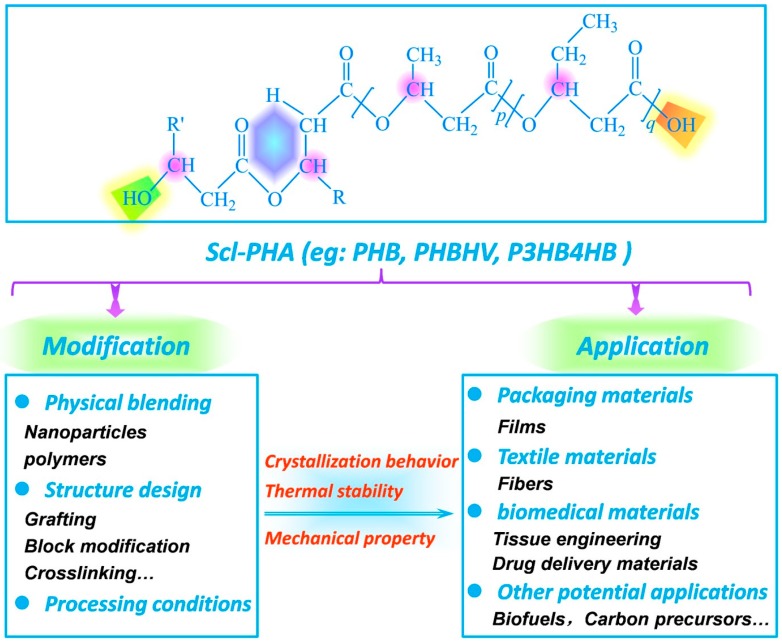
Overview of the modification and application of scl-PHA.

As summarized in Figure 13, this review offers a comprehensive overview on the modification of scl-PHA with low 3HV or 4HB content in terms of crystallization behaviors, thermal stability, and mechanical properties. The properties of scl-PHA can be improved to a certain extent by physical or chemical modification combined with the control of processing condition. Physical modification mainly refers to the blending of other polymers or nanoparticles with scl-PHA. By introducing polymers into scl-PHA, the production cost of scl-PHA can be significantly decreased, and the crystallization properties can also be controlled due to the interaction between additives and scl-PHA, which is a promising prospect for industrialization. Nanoparticles are incorporated into scl-PHA to increase the nucleation density, improve the crystallization rate, and reduce the spherulite size of scl-PHA, while the size distribution, dispersion, color, and heat resistance of added nanoparticles should be considered sufficiently. Chemical modification methods (such as grafting, blocking, and crosslinking) can be used to tailor the chemical structure of scl-PHA by controlling the interaction between the molecular chains of scl-PHA and the incorporated components, whereas the production costs of scl-PHA will be greatly increased and the introduced components may influence the original biodegradable properties of scl-PHA. In terms of processing conditions, scl-PHA fibers with excellent mechanical properties can be obtained via isothermal crystallization and a second drawing in ice water, which cannot achieve continuous spinning currently. However, the modified scl-PHA is still far from satisfactory partially due to the poor mechanical properties and the high production costs, leading to very few commercial products of scl-PHA available on the market. In order to expand the application of scl-PHA in the packaging, fiber, and biomedical fields, it is crucial to further enhance the properties of scl-PHA and decrease the production or modification costs, which will require the joint efforts of microbiologists, chemists, polymer scientists, engineers, and entrepreneurs. It is equally important to find a new application like carbon precursor for scl-PHA without any modification. We hope that the strategy of combining chemical, physical, and processing modifications can provide a train of thought for the development of bacterial products.

## Figures and Tables

**Table 1:** Degree of crystallinity, crystal structure, and *d* spacings of PHBHV samples. Reprinted with permission from [20]. Copyright (1989) American Chemical Society.

HV mol %	Crystallinity (%)	Crystal Structure	*d* Spacings (nm)			
			(020)	(110)	(002)	(211)
0	55 ± 5	P3HB	0.659	0.525	0.296	–
9	58 ± 5	P3HB	0.664	0.532	0.294	–
21	56 ± 5	P3HB	0.664	0.536	0.295	–
37	52 ± 5	P3HB	0.663	0.546	0.298	–
53	63 ± 5	P3HV	0.503	0.695	–	0.342
62	57 ± 5	P3HV	0.504	0.691	–	0.342
83	66 ± 5	P3HV	0.503	0.691	–	0.346
95	70 ± 5	P3HV	0.503	0.691	–	0.343

**Table 2:** Mechanical properties of scl-PHA fibers/ films.

Samples	*M*_w_	*PDI*	Processing methods	Tensile strength (MPa)	Young’s modulus (GPa)	Elongation (%)	Ref.
P3HB	3 × 10^5^	–	Gel spinning, three step stretch	360	5.6	37	[122]
	4.2 × 10^5^	1.35	Cold drawing, heat treatment	416	5.2	–	[124]
	3.72 × 10^6^	1.70	Two-step cold drawing, Annealing	1320	18.1	35	[123]
PHBHV (8% HV)	1 × 10^6^	2.8	Two-step cold drawing, Annealing	1065	8.0	40	[121]
